# CBX7 reprograms metabolic flux to protect against meningioma progression by modulating the USP44/c-MYC/LDHA axis

**DOI:** 10.1093/jmcb/mjad057

**Published:** 2023-10-03

**Authors:** Haixia Cheng, Lingyang Hua, Hailiang Tang, Zhongyuan Bao, Xiupeng Xu, Hongguang Zhu, Shuyang Wang, Zeyidan Jiapaer, Roma Bhatia, Ian F Dunn, Jiaojiao Deng, Daijun Wang, Shuchen Sun, Shihai Luan, Jing Ji, Qing Xie, Xinyu Yang, Ji Lei, Guoping Li, Xianli Wang, Ye Gong

**Affiliations:** Department of Pathology, School of Basic Medical Sciences, Fudan University, Shanghai 200032, China; Department of Neurosurgery, Huashan Hospital, Shanghai Medical College, Fudan University, Shanghai 200040, China; Department of Neurosurgery, Huashan Hospital, Shanghai Medical College, Fudan University, Shanghai 200040, China; Department of Neurosurgery, The First Affiliated Hospital of Nanjing Medical University, Nanjing 210000, China; Department of Neurosurgery, The First Affiliated Hospital of Nanjing Medical University, Nanjing 210000, China; Department of Pathology, School of Basic Medical Sciences, Fudan University, Shanghai 200032, China; Department of Pathology, School of Basic Medical Sciences, Fudan University, Shanghai 200032, China; Xinjiang Key Laboratory of Biology Resources and Genetic Engineering, College of Life Science and Technology, Xinjiang University, Urumqi 830046, China; Beth Israel Deaconess Medical Center, Harvard Medical School, Boston, MA 02215, USA; Department of Neurosurgery, University of Oklahoma Health Sciences Center, Oklahoma City, OK 73117, USA; Department of Neurosurgery, Huashan Hospital, Shanghai Medical College, Fudan University, Shanghai 200040, China; Department of Neurosurgery, Huashan Hospital, Shanghai Medical College, Fudan University, Shanghai 200040, China; Department of Neurosurgery, Huashan Hospital, Shanghai Medical College, Fudan University, Shanghai 200040, China; Department of Neurosurgery, Huashan Hospital, Shanghai Medical College, Fudan University, Shanghai 200040, China; Department of Neurosurgery, The First Affiliated Hospital of Nanjing Medical University, Nanjing 210000, China; Department of Neurosurgery, Huashan Hospital, Shanghai Medical College, Fudan University, Shanghai 200040, China; Fangshan Hospital of Beijing, University of Traditional Chinese Medicine, Beijing 102400, China; Center for Transplantation Science, Massachusetts General Hospital, Harvard Medical School, Boston, MA 02114, USA; Cardiovascular Research Center, Massachusetts General Hospital and Harvard Medical School, Boston, MA 02114, USA; School of Public Health, Shanghai Jiao Tong University School of Medicine, Shanghai 200025, China; Department of Neurosurgery, Huashan Hospital, Shanghai Medical College, Fudan University, Shanghai 200040, China; Department of Critical Care Medicine, Huashan Hospital, Shanghai Medical College, Fudan University, Shanghai 200032, China

**Keywords:** CBX7, meningioma, glycolysis, USP44, c-MYC, LDHA, malignancy, glucose metabolism

## Abstract

Meningioma is one of the most common primary neoplasms in the central nervous system, but no specific molecularly targeted therapy has been approved for the clinical treatment of aggressive meningiomas. There is hence an urgent demand to decrypt the biological and molecular landscape of malignant meningioma. Here, through the *in-silica* prescreening and 10-year follow-up studies of 445 meningioma patients, we uncovered that CBX7 expression progressively decreases with malignancy grade and neoplasia stage in meningioma, and a high CBX7 expression level predicts a favorable prognosis in meningioma patients. CBX7 restoration significantly induces cell cycle arrest and inhibits meningioma cell proliferation. iTRAQ-based proteomics analysis indicated that CBX7 restoration triggers the metabolic shift from glycolysis to oxidative phosphorylation. The mechanistic study demonstrated that CBX7 promotes the proteasome-dependent degradation of c-MYC protein by transcriptionally inhibiting the expression of a c-MYC deubiquitinase, USP44, consequently attenuates c-MYC-mediated transactivation of LDHA transcripts, and further inhibits glycolysis and subsequent cell proliferation. More importantly, the functional role of CBX7 was further confirmed in subcutaneous and orthotopic meningioma xenograft mouse models and meningioma patients. Altogether, our results shed light on the critical role of CBX7 in meningioma malignancy progression and identify the CBX7/USP44/c-MYC/LDHA axis as a promising therapeutic target against meningioma progression.

## Introduction

Meningioma is one of the most common primary neoplasms in the central nervous system with an estimated incidence of 7.86 cases per 100000 people per year (Karamitopoulou et al., 2010; Ostrom et al., 2015). Although ∼80% of meningiomas are benign and correspond to grade I according to the current World Health Organization (WHO) classification, up to 20% of cases show signs of increasing malignancy and belong to high-grade meningiomas (WHO grade II & grade III) (Preusser et al., 2018). Surgery followed by radiotherapy remains the mainstream strategy for treating meningioma in clinics, though this is insufficient to control 40%–75% of high-grade tumors (Rogers et al., 2015). Furthermore, incomplete resection due to some surgically inaccessible locations always leaves a major risk of meningioma regrowth and even malignant progression (Matias et al., 2019). In the past few years, tremendous efforts were made to decrypt the biological and molecular landscape of meningioma to explore novel signaling pathways that can be targeted against aggressive meningiomas (Bi et al., 2016; Angus et al., 2018; Du et al., 2018; Huang et al., 2019; Spiegl-Kreinecker et al., 2018), yet no molecularly targeted therapy has been approved for the clinical treatment of aggressive meningiomas by now. Therefore, comprehensively understanding the mechanistic basis of meningioma development is indispensable for discovering novel therapies to prevent meningioma progression and improve the prognosis of meningioma patients.

The abnormal metabolic shift from mitochondrial oxidative phosphorylation system (OXPHOS) to glycolysis, even in the presence of abundant oxygen, is a distinguished hallmark of solid tumors, known as the Warburg effect (Koppenol et al., 2011; Liberti and Locasale, 2016). Tumor cells that reprogram their metabolic flux to aerobic glycolysis will have significant growth advantages as they alter themselves to get enough adenosine triphosphate (ATP) without the limited supply of oxygen from the blood and alter the environment by acidosis to facilitate their growth and invasion through the destruction of adjacent normal populations, degradation of the extracellular matrix, and promotion of angiogenesis (Gatenby and Gillies, 2004). Clinically, rapid glucose consumption and lactate production have been associated with cancer aggressiveness and poor prognosis in patients with glioma, pancreatic cancer, melanoma, or many other tumors (Chan et al., 2016; Strickland and Stoll, 2017; Kim and DeBerardinis, 2019; Ruocco et al., 2019). Accumulated evidence shows that this metabolic switch is regulated by both oncogenes, such as Myc and AKT, and tumor suppressor genes, including p53 and PTEN, in different ways (Levine and Puzio-Kuter, 2010). For instance, c-Myc was reported to increase the transcription of several glucose transporters (GLUTs) and hexokinase 2, enhancing both uptake and retention of glucose by the cells (Dang, 1999). Blocking the metabolic switch from OXPHOS to aerobic glycolysis has been proven a promising strategy for cancer treatment (Akins et al., 2018). However, the knowledge about this metabolic switch in meningioma remains largely unknown.

Polycomb group (PcG) proteins, initially identified as a set of structurally diverse but functionally synergetic genes linking histone modifications with transcriptional repression, have long been linked to abnormal embryonic development and the occurrence of different forms of tumor (Richly et al., 2011). PcG proteins-associated multiple protein complexes are classified into two major functional groups, the Polycomb repressive complex I (PRC1) and complex II (PRC2). In mammalian cells, PRC2 mainly contributes to the establishment of histone-repressive marks, while PRC1 functions as a gene repressor by inhibiting the initiation of the transcription (Aranda et al., 2015). CBX7 is a chromobox family protein and canonically acts as a core component of PRC1. CBX7 was recognized by its capability to extend the lifespan of a wide range of normal human epithelial cells and immortalize mouse fibroblasts by repressing the INK4a/ARF tumor suppressor locus (Gil et al., 2004). Interestingly, CBX7 was initially identified as an oncogene in prostate cancer, lymphoma, and gastric cancer due to its inhibitory effect on INK4a/ARF locus (Bernard et al., 2005; Scott et al., 2007; Zhang et al., 2010), while several other studies reported the tumor suppressor roles of CBX7 in ovarian, thyroid, lung, pancreatic, and colon tumors (Pallante et al., 2008; Forzati et al., 2012b; Shinjo et al., 2014; Zheng et al., 2015). Notably, the role of CBX7 in meningioma is still unknown.

In this study, we found that CBX7 is downregulated during meningioma development and high CBX7 expression possesses significant prognostic value in meningioma patients. Functional studies revealed that CBX7 restoration significantly inhibits the proliferation of meningioma cells. iTRAQ-based comparative proteomics indicated that CBX7 restoration inhibits meningioma cell proliferation via reprogramming metabolic flux from glycolysis to OXPHOS. The targeted metabolic analysis further confirmed this metabolic switch by CBX7 restoration. In terms of mechanism, we found that CBX7 restoration suppresses the transcription of USP44, thus destabilizes c-MYC protein through proteasome-dependent degradation, and further results in the decreased transcription of a critical glycolysis enzyme, lactate dehydrogenase A (LDHA). *In vivo*, two xenograft animal models also confirmed the tumor suppressor role of CBX7 during meningioma progression. Taken together, we demonstrated that CBX7 inhibits LDHA transcription and subsequent glycolysis to protect against meningioma development through the destabilization of c-MYC protein.

## Results

### CBX7 expression progressively decreases with malignancy grade in meningioma

**Figure 1:**
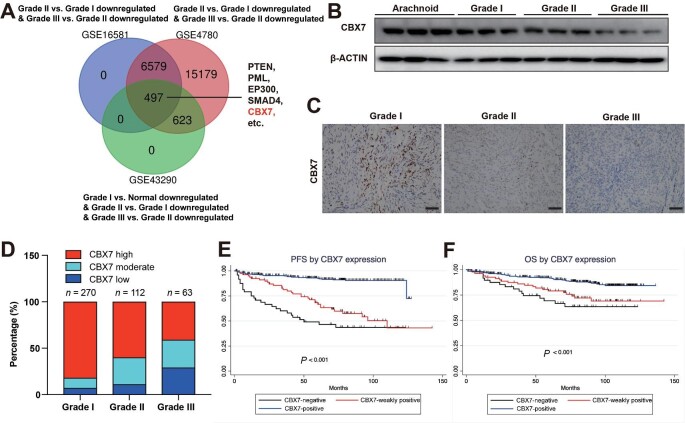
CBX7 expression progressively decreases with malignancy grade in meningioma and predicts the prognosis of meningioma patients. (**A**) Venn diagram of the repressed genes during the increase of malignancy grade of meningioma from three published transcriptome datasets. (**B**) Protein levels of CBX7 in meningiomas with different malignancy grades and normal arachnoid samples. (**C**) Representative immunohistology of CBX7 in meningiomas with different malignancy grades. Scale bar, 100 μm. (**D**) The correlation between CBX7 level and meningioma malignancy grade in 445 meningioma samples (*P* < 0.001, Fisher's exact test). (**E**) Progression-free survival of meningioma patients with different CBX7 levels (*P* < 0.001, log-rank test). (**F**) Overall survival of meningioma patients with different CBX7 levels (*P* < 0.001, log-rank test).

Meningioma is the most common primary neoplasm of the central neural system, accounting for >37.6% of primary brain tumors, and the incidence increases with aging (Achey et al., 2019; Ostrom et al., 2019). The lack of efficient molecularly targeted therapy results in an urgent need for advanced knowledge about the causes of intracranial meningioma. To find out potential tumor suppressors during meningioma development, we analyzed three published transcriptomes of meningiomas with different malignancy grades, GSE16581, GSE4780, and GSE43290. After extracting and overlapping the downregulated genes during every stage of meningioma development within three datasets, we identified 497 genes, including well-defined tumor suppressor genes PTEN, PML, EP300, and SMAD4 (Figure 1A). Strikingly, CBX7, which was known as an oncogene, was also indicated to be repressed during meningioma progression (Figure 1A). To confirm this finding, CBX7 expression in 12 flash-frozen surgical specimens, including normal arachnoid tissues and meningiomas with different malignancy grades, was examined by western blotting. The results demonstrated a gradual decrease in CBX7 expression as tumor pathological grade increased (Figure 1B). Next, we assessed the expression level of CBX7 in a larger cohort with 445 human meningioma samples, including 270 grade I, 112 grade II (atypical), and 63 grade III (anaplastic) meningiomas (Supplementary Table S1). The immunohistology data showed that >80% of grade I tumors exhibited high expression level of CBX7, while this percentage decreased as meningioma histological grade increased (Figure 1C and D). The statistical analysis demonstrated that CBX7 expression negatively correlated with the malignancy grade and Ki-67 index in meningioma (Figure 1D; Supplementary Table S1). These findings indicated that CBX7 functions as a tumor suppressor in meningioma.

### High CBX7 expression predicts a favorable prognosis for meningioma patients

To assess the predictive value of CBX7 expression level on the prognosis of meningioma patients, these 445 patients were followed up, and the progression-free survival (PFS) and overall survival (OS) were calculated. Through log-rank survival analysis, we found that along with tumor grade, tumor location, postoperative radiation, and tumor resection grade, CBX7 served as a positive prognostic factor for both PFS (Figure 1E) and OS (Figure 1F). Patients with CBX7-negative tumors showed the worst prognosis, while patients with CBX7-positive tumors showed prolonged survival. These findings indicated that CBX7 may act as a potent prognosis biomarker for meningioma patients.

### CBX7 restoration inhibits meningioma cell proliferation

**Figure 2:**
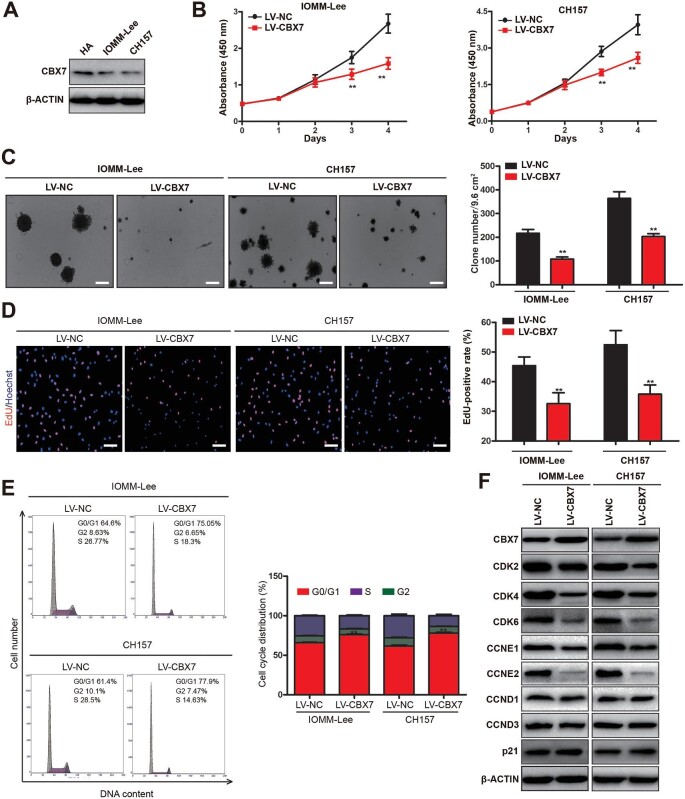
CBX7 restoration inhibits meningioma cell proliferation. (**A**) Protein levels of CBX7 in two meningioma cell lines (IOMM-Lee and CH157) and normal human arachnoid (HA) cells. (**B**–**F**) IOMM-Lee and CH157 meningioma cells with or without CBX7 restoration were examined. (**B**) MTS assay. (**C**) Colony formation assay. Scale bar, 200 μm. (**D**) EdU incorporation assay. Scale bar, 100 μm. (**E**) Cell cycle analysis. (**F**) Western blot analysis of protein levels of CDKs and cyclins. Data are shown as mean ± SD of three independent experiments. **P < 0.01 vs. the LV-NC group, Student *t*-test.

To investigate the functional role of CBX7 during meningioma development, two meningioma cell lines, IOMM-Lee and CH157, were used. CBX7 expression levels in these two cell lines were lower than that in normal arachnoid cells (Figure 2A). To evaluate the effect of CBX7 on cell proliferation, the cDNA of CBX7 was amplified from the human cDNA library, cloned into a lentivirus vector, and introduced into meningioma cells through lentivirus infection. Notably, CBX7 restoration significantly inhibited cell proliferation and the colony formation capability of both CH157 and IOMM-Lee cells (Figure 2B and C). The EdU incorporation assay also confirmed the inhibition of cell proliferation by CBX7 restoration in meningioma cells (Figure 2D).

Tumorigenesis is the result of uncontrolled cellular proliferation, which mainly results from cell cycle disorganization (Golias et al., 2004). Through flow cytometry, we found that CBX7 restoration substantially reduced the population of S/G2 phase cells and retarded the cells at the G0/G1 phase in both IOMM-Lee and CH-157 cells, suggesting a G1/S arrest (Figure 2E). Then, the expression levels of various cyclin-dependent kinases (CDKs) and cyclins were examined by western blotting. Interestingly, CBX7 restoration suppressed the expression of CDK2, CDK4, CDK6, CCNE1, and CCNE2 but had little effect on the levels of CCND1, CCND2, CCND3, p21, and p27 (Figure 2F). Collectively, these results suggested that CBX7 restoration inhibits cell proliferation and induces G1/S cell cycle arrest in meningioma cells.

### CBX7 restoration reprograms the metabolic flux from glycolysis to OXPHOS in meningioma cells

**Figure 3:**
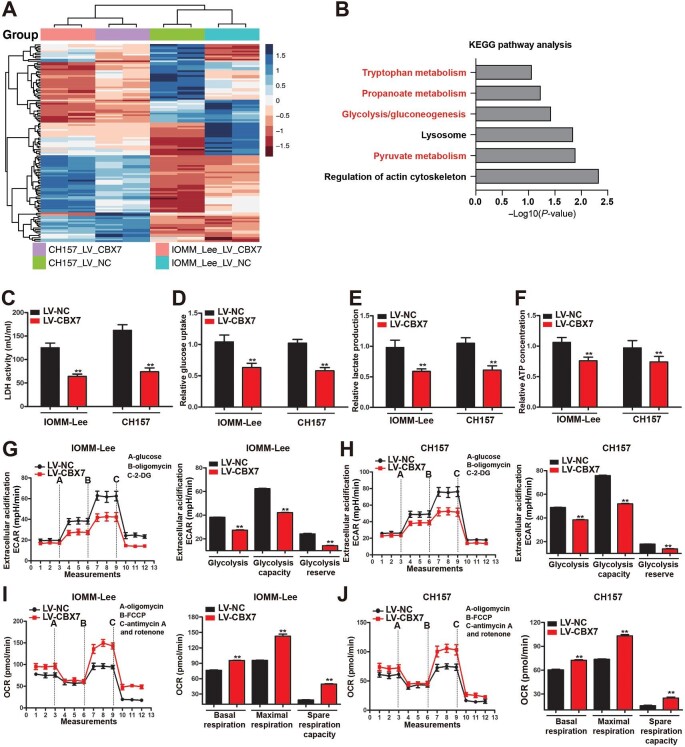
CBX7 restoration reprograms the metabolic flux from glycolysis to OXPHOS in meningioma cells. (**A**) Heatmap of differentially expressed proteins in IOMM-Lee and CH157 meningioma cells after CBX7 restoration. (**B**) KEGG pathway analysis of differentially expressed genes after CBX7 restoration. (**C**--**J**) IOMM-Lee and CH157 meningioma cells with or without CBX7 restoration were examined. (**C**–**E**) LDH activity (**C**), glucose uptake (**D**), and lactate production (**E**) in the conditional medium. (**F**) Intracellular ATP concentration. (**G** and **H**) Glycolytic activity analysis by monitoring ECAR. (**I** and **J**) OXPHOS activity analysis by monitoring OCR. Data are shown as mean ± SD of three independent experiments. **P < 0.01 vs. the LV-NC group, Student *t*-test.

Next, iTRAQ-based comparative proteomics analysis was performed to assess the proteome changes after CBX7 restoration in both IOMM-Lee and CH157 cells. A total of 4537 kinds of abundant protein were identified in the samples with the molecular weight arranged from 10 kDa to 300 kDa (Supplementary Table S2). Based on data acquisition, 121 genes were found differentially expressed in both IOMM-Lee and CH157 cells after CBX7 restoration (Figure 3A) and subjected to functional pathway analysis. The Kyoto Encyclopedia of Genes and Genomes (KEGG) annotation showed that these differentially regulated genes were mainly enriched in metabolism-related pathways, especially glycolysis and pyruvate metabolism (Figure 3B), which indicated that CBX7 restoration may influence glycolysis in meningioma cells.

Aerobic glycolysis is the primary characteristic of metabolic reprogramming in tumor cells, which is highly associated with increased progression and metastasis of tumors and thus considered a metabolic signature for malignant tumors (Gatenby and Gillies, 2004). During aerobic glycolysis, pyruvate derived from glucose is converted into lactate by LDH rather than subjected to the mitochondrial respiratory chain complex for OXPHOS (Vander Heiden et al., 2009). To elucidate the effect of CBX7 restoration on glucose metabolism, cellular LDH activity and glucose and lactate levels in conditioned cell culture media of control and CBX7-restored meningioma cells were measured. LDH activity, glucose consumption, and lactate secretion all decreased upon CBX7 restoration (Figure 3C–E), demonstrating an inhibited aerobic glycolysis. Although aerobic glycolysis is less efficient to generate ATP, it is faster than OXPHOS and accounts for 60% of total ATP production in cancer cells (Busk et al., 2008; Vander Heiden et al., 2009). The fluorometric assay-based ATP concentration quantification showed a significantly lower intracellular ATP content in CBX7-restored meningioma cells (Figure 3F). These results suggested that CBX7-restored meningioma cells might have shifted their metabolic flux from glycolysis to OXPHOS. To this end, glycolytic activities in meningioma cells with or without CBX7 restoration were assessed using the Seahorse XF Glycolysis Stress Test Kit. As shown in Figure 3G and H, the extracellular acidification rate (ECAR) was lower in CBX7-restored meningioma cells than in control cells with glucose supplementation, suggesting less glucose-dependent glycolysis. The maximal glycolytic capacity was measured by adding oligomycin to inhibit OXPHOS and showed a dramatic decrease after CBX7 restoration (Figure 3G and H). The glycolytic reserve was also significantly reduced in CBX7-restored meningioma cells when compared with control cells (Figure 3G and H). These findings manifested that CBX7 restoration attenuated the metabolic flux in maximal capacity to glycolytic pathways.

Furthermore, the mitochondrial oxygen consumption rate (OCR) was measured using a Seahorse XF Cell Mito Stress Test Kit, which can measure multiple key parameters of mitochondrial function after the sequential treatment of oligomycin (ATP synthase inhibitor), p-trifluoromethoxy carbonyl cyanide phenylhydrazone (FCCP; uncoupling agent), and antimycin A (Complex III inhibitor). As shown in Figure 3I and J, the basal respiration, ATP-linked respiration, maximal respiration, and spare respiration capacity were significantly higher in CBX7-restored meningioma cells than in control cells, suggesting an increased reliance on mitochondrial OXPHOS for energy production in CBX7-restored meningioma cells. Taken together, our discoveries demonstrated that CBX7 restoration reprograms the metabolic flux from glycolysis to OXPHOS in meningioma cells.

### CBX7 forces metabolic switch and inhibits cell proliferation through repressing LDHA

**Figure 4:**
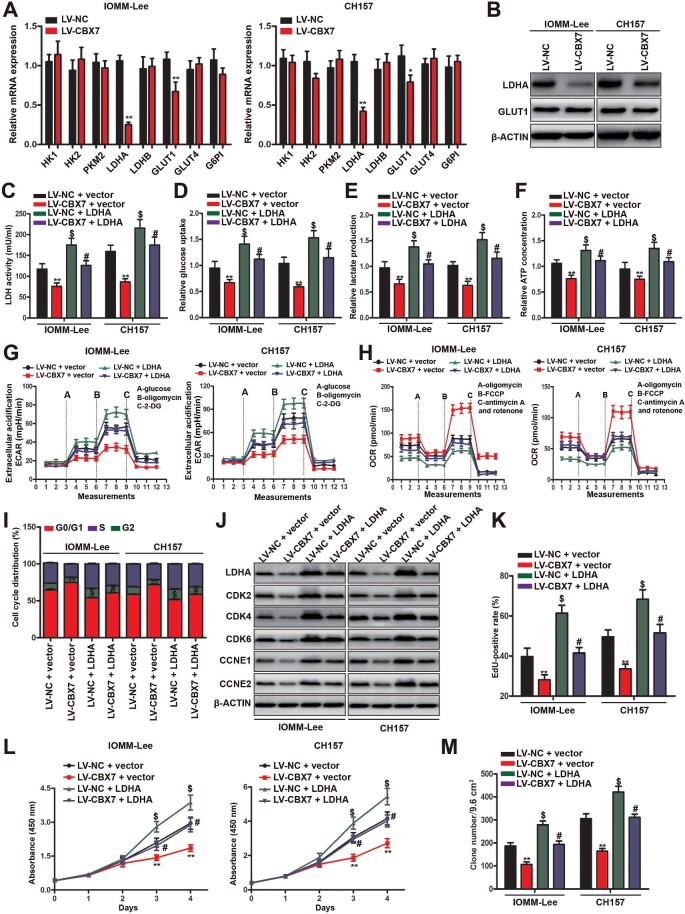
CBX7 triggers the metabolic switch by inhibiting LDHA transcription. (**A** and **B**) mRNA levels of critical glucose metabolism-related enzymes or glucose transporters (**A**) and protein levels of LDHA and GLUT1 (**B**) in IOMM-Lee and CH157 cells with or without CBX7 restoration. (**C**–**M**) CBX7-restored or control IOMM-Lee and CH157 meningioma cells with or without LDHA overexpression were examined. (**C**–**E**) LDH activity (**C**), glucose uptake (**D**), and lactate production (**E**) in the conditional medium. (**F**) Intracellular ATP concentration. (**G**) Glycolytic activity analysis by monitoring ECAR. (**H**) OXPHOS activity analysis by monitoring OCR. (**I**) Cell cycle analysis. (**J**) Western blot analysis of protein levels of CDKs and cyclins. (**K**) EdU incorporation assay. (**L**) MTS assay. (**M**) Colony formation assay. Data are shown as mean ± SD of three independent experiments. **P < 0.01 vs. the LV-NC + vector group, ^$^*P* < 0.05 vs. the LV-NC + vector group, ^#^*P* < 0.05 vs. the LV-CBX7 + vector group, Student *t*-test.

To explore the underlying mechanism of CBX7-triggered metabolic switch, we first examined the concomitant expression changes of key enzymes and glucose transporters in the glycolytic pathway, including HKI, HKII, PKM2, LDHA, LDHB, GLUT1, GLUT4, and G6PI. The quantitative polymerase chain reaction (qPCR) data showed that the expression of LDHA and GLUT1 transcripts was significantly inhibited by CBX7 restoration, while the expression levels of HKI, HKII, PKM2, LDHB, and GLUT4 remained unchanged (Figure 4A). Western blot analysis further confirmed the dramatic decrease of LDHA protein expression in CBX7-restored meningioma cells (Figure 4B), consistent with the proteomics data (Supplementary Table S2). To examine whether the declined LDHA expression is responsible for CBX7-forced metabolic switch, LDHA was overexpressed in meningioma cells with or without CBX7 restoration. As expected, the LDH activity was re-established in CBX7-restored meningioma cells with LDHA overexpression (Figure 4C). Subsequently, the hampered glucose uptake, lactate production, and intracellular ATP content by CBX7 restoration were recovered by ectopic LDHA expression (Figure 4D–F), indicating the recovered glycolytic activity. The measurements of ECAR and OCR further confirmed that the CBX7 restoration-induced metabolic switch from glycolysis to OXPHOS was reversed by the overexpression of LDHA (Figure 4G and H), which suggested that CBX7 restoration forces metabolic switch in meningioma cells via repressing LDHA expression.

Then, these meningioma cells were subjected to cell proliferation analysis. Strikingly, LDHA overexpression significantly accelerated cell cycle progression in CBX7-restored meningioma cells, which was proved by the enriched S/G2 phase population and higher expression of CDK and cyclin proteins (Figure 4I and J). The EdU incorporation assay and MTS cell proliferation assay further confirmed the retrieved cell proliferation by ectopic LDHA expression in CBX7-restored meningioma cells (Figure 4K and L). Moreover, LDHA overexpression also rescued the colony formation capacity of CBX7-restored meningioma cells (Figure 4M). Collectively, these results forcefully demonstrated that CBX7 inhibits cellular proliferation by inhibiting the transcription of LDHA.

### CBX7 enhances the proteasome-dependent degradation of c-MYC protein to repress LDHA transcription and glycolysis by transcriptionally inhibiting USP44 expression

**Figure 5:**
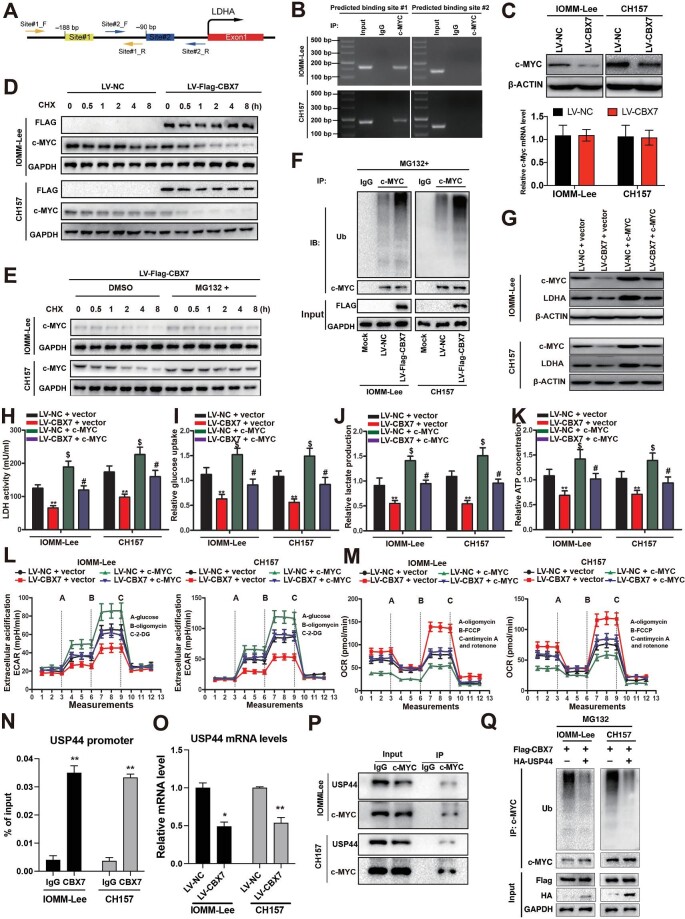
CBX7 destabilizes c-MYC protein by suppressing USP44 transcription to inhibit LDHA expression and glycolysis. (**A**) Schematic representation of two predicted c-MYC binding sites at the promoter of LDHA. (**B**) The binding of c-MYC at the promoter region of LDHA was validated by ChIP–qPCR. (**C**) Protein and mRNA levels of c-MYC in CBX7-restored or control IOMM-Lee and CH157 meningioma cells. (**D**) Protein levels of c-MYC in CBX7-restored or control IOMM-Lee and CH157 meningioma cells treated with 100 μg/ml CHX for different periods. (**E**) Protein levels of c-MYC in CBX7-restored IOMM-Lee and CH157 meningioma cells treated with 100 μg/ml CHX for different periods in the presence or absence of 10 μM MG132. (**F**) The ubiquitin modification level of c-MYC protein in CBX7-restored or control IOMM-Lee and CH157 meningioma cells after 10 μM MG132 treatment. (**G**–**M**) CBX7-restored or control IOMM-Lee and CH157 meningioma cells with or without c-MYC overexpression were examined. (**G**) Western blot analysis of protein levels of LDHA and c-MYC. (**H**–**J**) LDH activity (**H**), glucose uptake (**I**), and lactate production (**J**) in the conditional medium. (**K**) Intracellular ATP concentration. (**L**) Glycolytic activity analysis by monitoring ECAR. (**M**) OXPHOS activity analysis by monitoring OCR. (**N**) The binding of CBX7 at the promoter region of USP44 was validated by ChIP–qPCR. (**O**) CBX7 restoration suppresses the transcription of USP44 in IOMM-Lee and CH157 meningioma cells. (**P**) USP44 directly interacts with c-MYC protein in both IOMM-Lee and CH157 meningioma cells. (**Q**) USP44 deubiquitinates c-MYC protein in CBX7-overexpressing IOMM-Lee and CH157 meningioma cells. Data are shown as mean ± SD of three independent experiments. **P < 0.01 vs. the LV-NC + vector (or LV-NC) group, *P < 0.05 vs. the LV-NC group, ^$^*P* < 0.05 vs. the LV-NC + vector (or LV-NC) group, ^#^*P* < 0.05 vs. the LV-CBX7 + vector group, Student *t*-test.

Although CBX7 belongs to the repressive complex PRC1, it still needs specific transcription factors to facilitate its binding to the genome DNA (Grossniklaus and Paro, 2014). To identify the specific transcription factor involved in the repression of LDHA transcription, the promoter of LDHA was subjected to the *in-silica* prediction of potential binding factors. c-MYC, which has been reported to be involved in the regulation of glycolysis (Dang, 2013), was one of the top 10 possible binding factors (Supplementary Table S3) and has two predicted binding sites at the LDHA promoter (Figure 5A). Interestingly, c-MYC was reported to transactivate LDHA promoter in c-MYC-transformed human lymphoblastoid cells and Burkitt lymphoma cells (Shim et al., 1997). To validate this physical binding endogenously in meningioma cells, chromatin immunoprecipitation (ChIP) was performed using the c-MYC antibody in both IOMM-Lee and CH157 cells. The ChIP–qPCR data showed that c-MYC bound to the first predicted binding site, −180 bp upstream of the transcription start site, but not the second one in meningioma cells (Figure 5B). The protein levels of c-MYC in meningioma cells with or without CBX7 restoration were then measured by western blotting. Unexpectedly, c-MYC protein levels were remarkably lower in CBX7-restored meningioma cells than in control cells, while c-Myc mRNA levels in CBX7-restored and control cells were comparable (Figure 5C), suggesting that c-MYC protein is post-translationally modified by CBX7 restoration. Then, cycloheximide (CHX) was used to block *de novo* protein synthesis and the protein levels of c-MYC in meningioma cells with or without CBX7 restoration were measured. As shown in Figure 5D, c-MYC protein was rapidly degraded in CBX7-restored meningioma cells treated with CHX, while the degradation in control cells was much slower. To explore whether CBX7-induced c-MYC protein degradation depends on the proteasome, MG132 was utilized to inhibit the activity of the proteasome. Notably, the degradation of c-MYC protein by CBX7 restoration was hampered after MG132 treatment (Figure 5E). Usually, the protein is labeled with ubiquitin before being transported to the proteasome for degradation (Schrader et al., 2009). To further confirm the proteasome-mediated degradation of c-MYC, the ubiquitin level on c-MYC protein was examined by immunoprecipitating c-MYC protein after MG132 treatment and blotting with ubiquitin antibody. We found that CBX7 restoration dramatically increased the ubiquitin modification level on c-MYC protein in meningioma cells (Figure 5F). Therefore, our results demonstrated that CBX7 destabilizes c-MYC protein in a proteasome-dependent manner.

Next, we overexpressed c-MYC in meningioma cells with or without CBX7 restoration to determine the functional role of c-MYC in the CBX7/LDHA axis. As expected, c-MYC overexpression successfully rescued the decreased LDHA expression in CBX7-restored meningioma cells (Figure 5G). c-MYC overexpression also abrogated the inhibitory effect of CBX7 on LDH activity, lactate secretion, glucose consumption, and intracellular ATP levels (Figure 5H–K). Furthermore, the ECAR and OCR analyses suggested that the inhibited glycolysis and enhanced OXPHOS by CBX7 restoration were reversed upon c-MYC overexpression (Figure 5L and M). To explore how CBX7 regulates c-MYC protein stability, we analyzed the available CBX7 ChIP-seq dataset, GSE40740 (Pemberton et al., 2014), to identify potential ubiquitin/proteasome-related genes that are transcriptionally regulated by CBX7. Among the top putative CBX7 targets, a deubiquitinase, ubiquitin-specific peptidase 44 (USP44), has been shown to selectively remove K48-linked polyubiquitin chains from target proteins, such as MITA, FOXP3, and TRIM25, and protect them from proteasome-mediated degradation (Yang et al., 2020; Zhang et al., 2020; Chen et al., 2022). ChIP–qPCR with CBX7 antibody verified the binding of CBX7 at the promoter of USP44 in both meningioma cell lines (Figure 5N). Expectedly, qPCR data showed decreased USP44 mRNA levels upon CBX7 restoration (Figure 5O), demonstrating that CBX7 inhibits the transcription of USP44. We also identified USP44 protein in c-MYC-immunoprecipitated mixtures in both meningioma cell lines (Figure 5P). Then, HA-tagged USP44 was overexpressed in two CBX7-overexpressing meningioma cell lines. After pretreating the cells with MG132 to block the proteosome-mediated protein degradation, c-MYC protein was immunoprecipitated using the anti-c-MYC antibody and blotted with an anti-ubiquitin antibody. The results showed that USP44 overexpression reduced the levels of CBX7-mediated c-MYC ubiquitination in both IOMM-Lee and CH157 meningioma cells (Figure 5Q). Taken together, these data suggested that CBX7 restoration transcriptionally inhibits the expression of USP44, which fails to protect c-MYC protein from proteasome-mediated degradation through deubiquitinating c-MYC, and therefore leads to decreased LDHA transcription and subsequent aerobic glycolysis.

### CBX7 restoration suppresses the progression of both subcutaneous and orthotopic meningioma xenografts in mice

**Figure 6:**
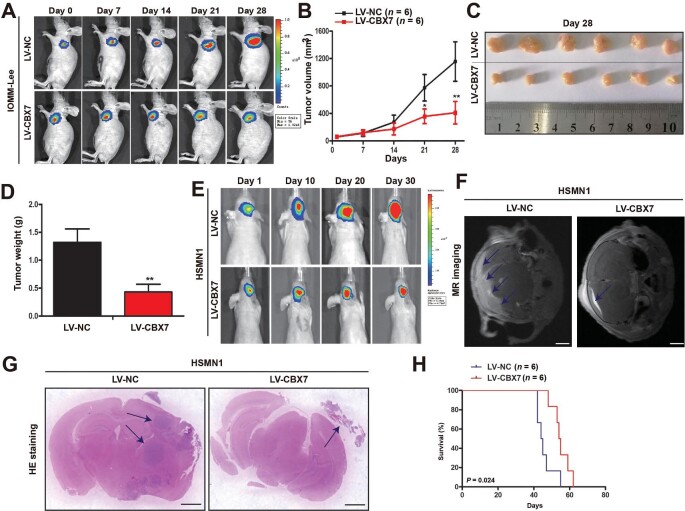
CBX7 restoration suppresses the progression of both subcutaneous and orthotopic meningioma xenografts in mice. (**A**–**D**) Subcutaneous xenograft experiments were conducted with IOMM-Lee meningioma cells with or without CBX7 restoration. (**A**) Bioluminescence-mediated non-invasive imaging of mice bearing subcutaneous xenografts. (**B**) Tumor volume of subcutaneous xenografts. (**C** and **D**) Tumor size (**C**) and weight (**D**) of subcutaneous xenografts on Day 28 after injection. (**E**–**H**) Orthotopic xenograft experiments were conducted with HSMN1 meningioma cells with or without CBX7 restoration. (**E**) Bioluminescence-mediated non-invasive imaging of mice bearing orthotopic xenografts. (**F**) MR imaging of orthotopic xenografts. Scale bar, 1 mm. (**G**) HE staining of orthotopic xenografts. Scale bar, 1 mm. (**H**) Survival curve of mice bearing orthotopic xenografts. Data are shown as mean ± SD from 6 mice per group. **P < 0.01 vs. the LV-NC group, Student *t*-test.

To recapitulate the functional role of CBX7 during meningioma progression, we subcutaneously injected IOMM-Lee meningioma cells with or without CBX7 restoration to build the subcutaneous meningioma xenograft mouse model. The meningioma xenograft growth was monitored weekly after injection by both bioluminescence imaging and tumor volume measurement. As expected, CBX7 restoration significantly impeded the progression of subcutaneous meningioma xenograft in mice (Figure 6A–D). To mimic the meningioma growth niche *in vivo*, the orthotopic meningioma xenograft mouse model was built by intracranially injecting HSMN1 primary meningioma cells, which were derived from grade III meningioma patients. Consistently, CBX7-restored HSMN1 cells showed a slower growth in the cranium when compared with control cells (Figure 6E). Both magnetic resonance (MR) imaging and necropsy further confirmed the tumor-suppressive role of CBX7 in meningioma xenografts (Figure 6F and G). More importantly, the survival period of mice bearing CBX7-restored meningioma xenofrafts was significantly prolonged (Figure 6H). These results demonstrated that CBX7 restoration suppresses meningioma progression *in vivo*.

### The CBX7/c-MYC/LDHA axis in meningioma patients

**Figure 7:**
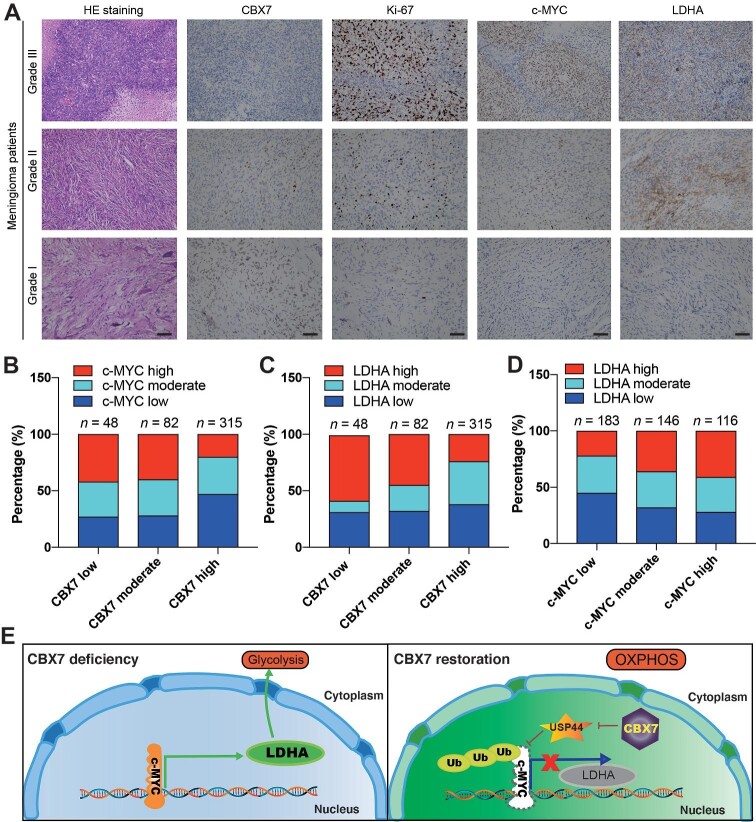
The validation of the CBX7/c-MYC/LDHA axis in human meningioma patients. (**A**) Representative images of HE staining and immunostaining of CBX7, Ki67, c-MYC, and LDHA in meningiomas with different malignancy grades. Scale bar, 100 μm. (**B**) The correlation between CBX7 and c-MYC in 445 meningioma patients (*P* < 0.001, Fisher's exact test). (**C**) The correlation between CBX7 and LDHA in 445 meningioma patients (*P* < 0.001, Fisher's exact test). (**D**) The correlation between c-MYC and LDHA in 445 meningioma patients (*P* = 0.002, Fisher's exact test). (**E**) Schematic representation of the functional role of CBX7 in inhibiting glycolysis and proliferation of meningioma cells by suppressing USP44 transcription, destabilizing c-MYC protein, and sequestering c-MYC from the LDHA promoter.

To determine the clinical significance of the CBX7/c-MYC/LDHA axis in meningioma, immunohistochemistry was performed in meningioma patient samples with different malignancy grades to detect the expression levels of CBX7, Ki-67, c-MYC, and LDHA. Consistently, the stronger immune reactivity of CBX7 was significantly associated with the lower c-MYC expression (Figure 7A). Besides, CBX7 expression negatively correlated with LDHA and Ki-67 expression, while c-MYC expression positively correlated with LDHA and Ki-67 expression (Figure 7B–D). Collectively, these data supported the functional role of the CBX7/c-MYC/LDHA axis during meningioma progression.

## Discussion

CBX7 plays a critical role in PRC1-mediated epigenetic repression of gene transcription. In this study, we uncovered that CBX7 is suppressed in malignant meningioma and CBX7 restoration significantly attenuates meningioma progression and prolongs survival. Mechanistically, CBX7 restoration destabilizes c-MYC protein by transcriptionally suppressing USP44 expression and then inhibits the c-MYC-dependent transactivation of LDHA transcription, thus inhibiting glycolysis activity and subsequent cell proliferation in meningioma cells (Figure 7E). More importantly, the role of the CBX7/USP44/c-MYC/LDHA axis is confirmed in human meningioma tissue samples.

CBX7 was initially identified to be involved in overcoming cellular senescence and extending the lifespan of human cells (Gil et al., 2004). Recently, accumulated evidence has demonstrated that CBX7 plays versatile functions in tumorigenesis; however, the role of CBX7 during tumor development remains inconsistent in different cancers. Loss of CBX7 expression has been shown to correlate with a highly malignant phenotype and poor prognosis in patients with hepatocellular carcinoma (Zhu et al., 2019), glioma (Yu et al., 2017), pancreatic cancer (Karamitopoulou et al., 2010), breast cancer, thyroid cancer (Pallante et al., 2008), cervical cancer (Li et al., 2019), ovarian cancer (Shinjo et al., 2014), and colon cancer (Zheng et al., 2015), which supports the tumor-suppressive role of CBX7. Meanwhile, several studies also demonstrated an oncogenic role of CBX7 in multiple types of human cancers, including prostate cancer (Bernard et al., 2005), lymphoma (Scott et al., 2007), and gastric cancer (Zhang et al., 2010). Nevertheless, the role of CBX7 during meningioma progression remains to be elucidated. We found that CBX7 expression progressively decreases with malignancy grade and neoplasia stage in meningioma and high CBX7 expression predicts a favorable prognosis in meningioma patients. CBX7 restoration significantly inhibits meningioma cell proliferation and extends the survival of mice bearing orthotopic meningioma xenografts. Therefore, our results support the tumor-suppressive role of CBX7 during meningioma development.

Numerous studies have shown that cancer cells prefer glycolysis for energy production to meet the enormous demand for ATP, while normal cells mostly rely on OXPHOS (Gatenby and Gillies, 2004; Vander Heiden et al., 2009). This metabolic switch, known as the ‘Warburg Effect’, has been considered one of the most fundamental metabolic alterations during malignant transformation and one of the most distinctive hallmarks of cancer cells (Hanahan and Weinberg, 2011). Therefore, inhibiting glycolysis is suggested to have promising anticancer potential. Indeed, inhibition of lactate production through targeting LDHA has shown significant benefits in multiple cancers (Feng et al., 2018), including melanoma, lung cancer, breast cancer, cervical cancer, leukemia, glioma, and adrenal cancer. However, whether targeting glycolysis can inhibit meningioma progression remains unknown. Through iTRAQ-based proteomics analysis, we found that CBX7 restoration switches the metabolic flux from glycolysis to OXPHOS in meningioma cells. A ‘small-scale’ screening of key genes responsible for glucose metabolism or glucose transportation identified LDHA as the most significantly regulated target of CBX7. Strikingly, LDHA overexpression blocked CBX7 restoration-triggered metabolic shift and subsequent cell proliferation suppression. Taken together, our data suggest that targeting glycolysis via inhibiting LDHA can suppress meningioma cell proliferation.

CBX7 belongs to the PcG protein family and functions as a PRC1 component. CBX7 could differentially modulate the expression of crucial genes involved in cancer progression due to its ability to bind to chromatin and recruit PRCs (Forzati et al., 2012a; Pallante et al., 2014). Interestingly, CBX7 was previously reported to synergize with c-MYC to promote the growth of prostatic cancer cells or cooperate with c-MYC to produce highly aggressive B cell lymphomas, acting as an oncogene and positively correlating with c-MYC (Bernard et al., 2005; Scott et al., 2007). Nonetheless, we unveiled that CBX7 expression level negatively correlates with c-MYC level in meningioma patients and CBX7 can destabilize c-MYC protein in a proteasome-dependent manner. CBX7 restoration inhibits the transcription of a deubiquitinase, USP44, which then fails to protect c-MYC protein from proteasome-dependent degradation. Importantly, c-MYC directly binds to the LDHA promoter to activate its transcription in malignant meningioma cells, while CBX7 restoration forces the degradation of c-MYC protein and then attenuates c-MYC-mediated transactivation of LDHA transcription. Although the details regarding the specific E3 ubiquitin ligase that targets c-MYC protein for degradation remain to be further elucidated, the present study indicates a novel function of the CBX7/USP44/c-MYC pathway in meningioma.

There are still several limitations in the present study. First, we only used two representative malignant meningioma cell lines, IOMM-Lee without NF2 mutation and CH157 with NF2 mutation, to explore the regulation and roles of the CBX7/USP44/c-MYC/LDHA axis. Although another primary meningioma cell line, HSMN1, was used in the animal model and similar phenotypes were observed, we cannot exclude the existence of other unknown confounders that might affect the conclusion regarding the regulation of the CBX7/USP44/c-MYC/LDHA axis. In addition, we only correlated the expression patterns of CBX7, c-MYC, LDHA, and Ki-67 in 445 meningioma patient samples with the follow-up outcomes of these patients. The therapeutical effectiveness of targeting the CBX7/USP44/c-MYC/LDHA axis in meningioma patients needs to be further explored. Second, we only screened a small-scale gene set for glucose uptake and metabolism to identify LDHA as the target responsible for CBX7-forced metabolic switch. Previous studies reported that c-MYC can directly transactivate the transcription of LDHA gene (Shim et al., 1997), which was confirmed in meningioma cells in this study. c-MYC has been reported as ‘a master regulator’ of cellular metabolism in tumors by affecting multiple signaling pathways or genes (Miller et al., 2012). Since we did not analyze the transcriptome and chromatin binding patterns of CBX7 or c-MYC in meningioma cells with or without CBX7, we cannot exclude the possibility that c-MYC or CBX7 regulates other pathways to mediate the metabolic switch and inhibit cell proliferation. Third, although we identified a c-MYC deubiquitinase, USP44, we did not identify the specific E3 ligase that directly targets c-MYC protein to proteasomes.

In summary, this study provides both clinical and mechanistic evidence supporting the functional role of the CBX7/USP44/c-MYC/LDHA axis in regulating meningioma progression through reprogramming glucose metabolism from glycolysis to OXPHOS, shedding light on a previously unexpected role of CBX7 in meningioma progression and a potential therapeutic strategy for high-grade meningioma patients.

## Materials and methods

### Patients and samples

We performed a retrospective cohort review study of 445 patients who underwent surgical resection of intracranial meningioma in the main branch of Huashan Neurosurgical Center in the period of 2003–2013. All the surgical specimens were reviewed and confirmed by two experienced neuro-pathologists independently according to the newest 2016 WHO grading criterion for meningioma. Patients’ data were retrieved from electronic and printed medical records. Follow-up was conducted routinely mainly through the out-patient department. This study was approved by the institutional ethics review board of Huashan Hospital, Fudan University (KY-2017-09), and informed consent was acquired from all the involved patients.

### Immunohistochemistry

Antigen retrieval was performed by heating the tissue slides in the citrate buffer. Immunostaining was performed using anti-CBX7 antibody (Abcam, ab21873; 1:400), anti-c-MYC antibody (Abcam, ab32072; 1:400), anti-LDHA antibody (Cell Signaling Technology, #3582S; 1:400), and anti-Ki-67 antibody (Abcam, ab15580; 1:400), followed by detection using the EnVision+ System-HRP (Dako). Appropriate positive and negative controls were included in each run of immunostaining. Two experienced neuro-pathologists independently evaluated and scored the tissue slides according to the intensity and extent of staining. The percentage of CBX7-positive cancer cells was scored on a scale of 0–2 (0 = low staining, 1 = moderate staining, 2 = high staining).

### Cell lines and culture conditions

The cell line IOMM-Lee was courtesy of Dr Randy Jensen (University of Utah) and the cell line CH157 was courtesy of Dr Yancey Gillespie (University of Alabama-Birmingham). Both cell lines were cultured in Dulbecco's Modified Eagle F12 Medium supplemented with 10% fetal bovine serum and 2 mM L-Glutamine (Life Technology). Both cell lines were used within 20 passages and thawed fresh every 2 months. Primary meningioma cell line, HSMN1, was derived as described previously (Tang et al., 2017). Briefly, fresh meningioma surgical specimens were washed in phosphate-buffered saline, digested in a mixture of collagenase (1 mg/ml) and dispase (2 mg/ml). The primary cells were maintained in sterile, serum-free NeuroCult Basal Medium (Stem Cell Technologies) with NeuroCult Proliferation Supplement (Stem Cell Technologies) as well as 20 ng/ml basic fibroblast growth factor (Peprotech) and 20 ng/ml epidermal growth factor (Peprotech). The cells were passaged every 5 days by dissociating with TrypLE (Thermo Fisher Scientific). All *in vitro* experiments were performed with three biological replicates.

### Lentivirus packaging and infection

The entire coding sequences of CBX7, LDHA, USP44, and c-MYC were obtained from the human umbilical vein endothelial cell cDNA library by reverse transcription (RT)–PCR and cloned into pHAGE-CMV-MCS-PGK-3×Flag and pCMV-HA vectors. Lentivirus packaging and infection were performed as described previously (Li et al., 2017). Briefly, HEK293T cells were incubated with the transfection mix overnight, and then the medium was replaced by fresh medium. After 48 h, the conditional medium containing lentiviral particles was collected and filtered through a 0.45-μm filter. Stable cell lines were established by incubating IOMM-Lee, CH157, and HSMN1 cells in the lentivirus-containing conditional medium with polybrene (8 μg/ml) for 8 h. Two weeks later, cells were selected with puromycin (10 ng/ml) for 5 days.

### Western blot analysis

Cells were lysed in immunoprecipitation buffer (Beyotime) with a cocktail of protease and phosphatase inhibitors (Roche) and centrifuged at 12000 rpm for 25 min. Protein samples (20 μg) were separated on 4%–20% SDS–PAGE gels and transferred to polyvinylidene difluoride membranes by electroblotting. After blocking with 5% nonfat milk in TBST (20 mM Tris–HCl, pH 7.5, 150 mM NaCl, 0.1% Tween 20), membranes were incubated at 4°C overnight with primary antibodies against CBX7 (Abcam, ab21873), c-MYC (Abcam, ab32072), LDHA (Cell Signaling Technology, #3582), CDK2 (Abcam, ab32147), CDK4 (Abcam, ab108357), CDK6 (Abcam, ab54576), CCNE1 (Abcam, ab33911), CCNE2 (Abcam, ab32103), CCND1 (Cell Signaling Technology, #2922), CCND3 (Cell Signaling Technology, #2936), P21 (Cell Signaling Technology, #2946), FLAG (Cell Signaling Technology, #14793), HA (Cell Signaling Technology, #3724), Ubiquitin (Abcam, ab134953), and β-ACTIN (Affinity Biosciences, #T0022). Horseradish peroxidase-conjugated secondary antibodies were then used and the specific blots were detected by ECL reagent (Amersham Bioscience).

### Measurements of ECAR and OCR

ECAR and OCR were measured on the Seahorse XF96 Extracellular Flux Analyzer (Seahorse Bioscience) by using the Seahorse XF Glycolysis Stress Test Kit (Agilent, 103020-100) and Seahorse XF Cell Mito Stress Test Kit (Agilent, 103015-100), respectively. Briefly, IOMM-Lee and CH157 cells were plated in XF96 cell culture plates at a density of 1 × 10^5^ cells per well and incubated for 72 h at 37°C with 5% CO_2_. After baseline measurements, glucose, the oxidative phosphorylation inhibitor oligomycin, and the glycolytic inhibitor 2-DG were sequentially injected into each well at the indicated time points for ECAR measurement, while oligomycin, the uncoupler FCCP, the mitochondrial Complex I inhibitor rotenone, and the mitochondrial Complex III inhibitor antimycin A (Rote/AA) were sequentially injected for OCR measurement. Each time point included 5 min of rest, 1 min of mixing, and 3 min of measuring. The results (OCR in pmol/min and ECAR in mpH/min) were normalized to cell counts and then compared using *t*-tests in Prism.

### RT–PCR and qPCR

Total RNA was extracted using TRIzol (Thermo Fisher Scientific) as described previously (Li et al., 2022) and reverse-transcribed using a cDNA synthesis kit (Quanta-Bioscience). qPCR was carried out using SYBR Green I PCR Master Mix (Roche) on the Roche LightCycler 480 system. The gene expression level was normalized to the housekeeping gene *GAPDH*. All the primers used are listed in Supplementary Table S4.

### Cell viability analysis

Meningioma cell proliferation was estimated using CCK-8 (Beyotime, C0037). IOMM-Lee and CH157 cells with or without CBX7 restoration were seeded into 96-well cell culture plates. Every well contained 2 × 10^3^ cells in 100 μl culture media. After 24, 48, 72, or 96 h, the medium in each well was replaced with 100 μl fresh medium with 10% CCK-8, and the cells were incubated at 37°C for an additional 2 h. The absorbance was measured at 450 nm wavelength.

### Colony formation assay

IOMM-Lee and CH157 cells with or without CBX7 restoration were counted and plated at a density of 400 cells per well for colony formation in 6-well plates. The cells were incubated at 37°C for 2 weeks with growth media being replaced every 4 days. Colonies were fixed in chilled methanol for 30 min, followed by staining with 0.5% crystal violet for another 5 min.

### Glucose uptake and lactate production

Cells were seeded in 12-well plates at a density of 5 × 10^4^ cells per well. Culture media was collected at 48 h and stored at −20°C until assayed. Glucose uptake was detected using the Quantichrom Glucose Assay kit (BioAssay Systems, DIGL-100) by measuring the absorbance at 630 nm on the Synergy H4 Hybrid Multi-Mode Microplate Reader (BioTek). Lactate production was detected using the Enzychrom L-Lactate Assay kit. Both results were normalized to total protein.

### Measurement of intracellular ATP

Intracellular ATP was measured using an ATP assay kit (Beyotime, S0026) according to the manufacturer's protocol. Briefly, 1 × 10^6^ cells were seeded in each well of a 6-well plate and lysed after 48 h. The cellular ATP was normalized to total protein content. Experiments were performed in triplicate.

### Measurement of cellular LDH activity

Cellular LDH activity was measured using a Lactate Dehydrogenase Assay Kit (Colorimetric) (Abcam, ab102526) according to the manufacturer's protocol. Briefly, 1 × 10^6^ cells were lysed in 1 ml lysis buffer and then subjected to the LDH assay. The cellular LDH activity was normalized to total cell lysate volume. Experiments were performed in triplicate.

### EdU proliferation assay

Click-iT EdU Alexa Fluor 594 Imaging Kit (Thermo Fisher Scientific, C10339) was used according to the manufacturer's protocol. Briefly, 1 × 10^6^ cells were seeded in each well of a 6-well plate for 48 h and then fed with EdU (10 μM) for additional 6 h. Experiments were performed in triplicate.

### ChIP assay

IOMM-Lee or CH157 meningioma cells (2 × 10^6^) were prepared for the ChIP assay using a kit (Millipore, 17-295) according to the manufacturer's protocol. Briefly, cells were cross-linked with 1% formaldehyde at room temperature for 10 min. The DNA–protein complexes were immunoprecipitated with anti-c-MYC antibody (Abcam, ab32072) or anti-CBX7 antibody (Thermo Fisher Scientific, PA5-40905) and protein A/G-agarose (Millipore). The resulting precipitated DNA samples were then amplified by PCR using primers specific for the LDHA promoter. The following primers were used: predicted binding site #1: 5′-ATGGATGAGGAAACTGAAGGTCG-3′ (forward) and 5′-GGGTGGATGCCGAGCCAG-3′ (reverse); predicted binding site #2: 5′-GGTCGGTTGTCTGGCTGC-3′ (forward) and 5′-AGTGGAACAGCTATGCTGACGTCAG-3′ (reverse). The PCR products were resolved electrophoretically on a 2% agarose gel and visualized using ethidium bromide staining. For CBX7 ChIP–qPCR, precipitated DNA samples were amplified by qPCR using USP44 promoter primers: 5′-CGCCAGGACTTTTCACTTAGG-3′ (forward) and 5′-CAACACTTGAAGCATCTGAAAAC-3′ (reverse).

### Xenograft mouse models

For subcutaneous xenograft studies, cells (1 × 10^7^) were inoculated into the right flank of 4–6-week-old severe combined immunodeficient (SCID) mice. Tumor volume (V) was monitored by measuring tumor length (L) and width (W) with calipers and then calculated with the formula: (L × W^2^) × 0.5. For meningioma orthotropic xenograft survival experiments, 2 × 10^5^ IOMM-Lee/vector or IOMM-Lee/CBX7 cells were stereotactically implanted into the frontal subdural region (2.5 mm lateral from bregma and 1.0 mm deep from the skull) of SCID mice. For HSMN1 xenograft experiments, 5 × 10^6^ cells were used for each mouse. The mice were imaged once a week using the IVIS200 imaging system (Xenogen Corporation). D-Luciferin potassium salt (VivoGlo Luciferin, Promega) was injected intraperitoneally (300 mg/kg) and the mice were anesthetized in an oxygen-rich induction chamber with 2% isoflurane (Abbie). Images were quantified using Living Image Software (Xenogen). The mice were monitored for status twice per week and were sacrificed when neurologic deficits or general conditions reached the criteria for euthanasia. Brains were removed and frozen immediately at −80°C for pathological studies. All mouse procedures were approved by the Committee on Research Animal Care at Shanghai Medical College, Fudan University.

### Statistical analysis

All analyses were performed with Stata 13.3 software (StataCorp). Continuous features were described as mean ± standard deviation (SD) and analyzed with Student *t-*test or Mann–Whitney *U* test, while categorical variables were analyzed with either Pearson chi-square test or Fisher's exact test. A log-rank test was applied for survival analysis. All *in vitro* and *in vivo* experiments, including cell viability assay, metabolite quantification, and western blotting, were performed at least three times to confirm reproducibility. *P *< 0.05 was considered statistically significant.

## Supplementary Material

mjad057_Supplemental_Files

## Notes

**Author contributions:** H.C., L.H., H.T., Z.B., and X.X. conducted most of the experiments, wrote a draft of the manuscript, and contributed significantly to the revision. H.Z., S.W., Z.J., R.B., I.F.D., J.D., D.W., S.S., S.L., J.J., X.Y., J.L., and Q.X. discussed the manuscript extensively and contributed to project conception, design, and revision. J.L., G.L., X.W., and Y.G. conceived the project, analyzed the data, and wrote the manuscript. X.W. and Y.G. provided significant financial support. All authors discussed the experiments and contributed to the text of the manuscript.
