# Supplementary material for: CBX7 reprograms metabolic flux to protect against meningioma progression by modulating the USP44/c-MYC/LDHA axis
Source: J Mol Cell Biol. 2023 Oct 3;15(10):mjad057. doi: 10.1093/jmcb/mjad057 (PMC11195615; doi:10.1093/jmcb/mjad057)
Supplement: mjad057_Supplemental_Files [file mjad057_supplemental_files.zip › JMCB-2022-0008.R3_Supplementary material.pdf]

Supplementary material

CBX7 reprograms metabolic flux to protect against meningioma progression by modulating the USP44/c-MYC/LDHA axis

Haixia Cheng, Lingyang Hua, Hailiang Tang, Zhongyuan Bao, Xiupeng Xu, Hongguang Zhu, Shuyang Wang, Zeyidan Jiapaer, Roma Bhatia, Ian F. Dunn, Jiaojiao Deng, Daijun Wang, Shuchen Sun, Shihai Luan, Jing Ji, Qing Xie, Xinyu Yang, Ji Lei, Guoping Li, Xianli Wang, and Ye Gong

Supplementary Table S1 Clinical characteristics and their correlations with CBX7 expression.

**Variable**	**CBX7 low**	**CBX7 moderate**	**CBX7 high**	***P***
Age				
<60	10 (20.8%)	25 (30.5%)	98 (31.1%)	0.348
≥60	38 (79.2%)	57 (69.5%)	217 (68.9%)	
Gender				
Male	13 (27.1%)	34 (41.5%)	115 (36.5%)	0.259
Female	35 (72.9%)	48 (59.5%)	200 (63.5%)	
WHO grade				
I	18 (37.5%)	30 (36.6%)	222 (70.5%)	0.0001*
II	12 (25%)	33 (40.2%)	67 (21.3%)	
III	18 (37.5%)	19 (23.2%)	26 (8.2%)	
Tumor location				
Skull-base	26 (54.2%)	60 (73.2%)	200 (63.5%)	0.081
Non-skull base	22 (45.8%)	22 (26.8%)	115 (36.5%)	
Tumor recurrent status				
De novo	22 (45.8%)	50 (61.0%)	289 (91.7%)	0.004*
Recurrent	26 (54.2%)	32 (39.0%)	26 (9.3%)	
Preoperative KPS				
<80	18 (37.5%)	29 (35.3%)	272 (86.3%)	0.000*
≥80	30 (62.5%)	53 (64.7%)	43 (13.9%)	
Simpson resection grade				
GTR	34 (70.8%)	72 (87.8%)	287 (91.1%)	0.0003*
STR	14 (29.2%)	10 (12.2%)	28 (9.9%)	
Ki-67 labeling index				
<3	18 (37.5%)	42 (51.2%)	197 (62.5%)	0.0071*
≥3	30 (62.5%)	40 (49.8%)	118 (37.5%)	

PR, progesterone receptor; Coef., coefficient; CI, confidence interval; *$P < 0.05$.

Supplementary Table S2 iTRAQ-based proteomics analysis of meningioma cells with or without CBX7 restoration. (see Excel)

Supplementary Table S3 Predicted top 10 transcription factors bound to the LDHA promoter.

1	EP300
2	ZBTB40
3	SIN3A
4	NRF1
5	c-MYC
6	TCF12
7	POLR2G
8	USF1
9	SP1
10	NCOR1

Supplementary Table S4 Primers used for qPCR.

	**Forward sequence**	**Reverse sequence**
GAPDH	TGTGGGCATCAATGGATTTGG	ACACCATGTATTCCGGGTCAAT
LDHA	ATCTTGACCTACGTGGCTTGGA	CCATACAGGCACACTGGAATCTC
LDHB	GGACAAGTTGGTATGGCGTGTG	AAGCTCCCATGCTGCAGATCCA
HK1	CTGCTGGTGAAAATCCGTAGTGG	GTCCAAGAAGTCAGAGATGCAGG
HK2	GAGTTTGACCTGGATGTGGTTGC	CCTCCATGTAGCAGGCATTGCT
CBX7	CATGGAGCTGTCAGCCATC	CTGTACTTTGGGGGCCATC
PKM2	ATGGCTGACACATTCCTGGAGC	CCTTCAACGTCTCCACTGATCG
GLUT1	TTGCAGGCTTCTCCAACTGGAC	CAGAACCAGGAGCACAGTGAAG
GLUT4	CCATCCTGATGACTGTGGCTCT	GCCACGATGAACCAAGGAATGG
G6PI	AGGCTGCTGCCACATAAGGT	AGCGTCGTGAGAGGTCACTTG
c-MYC	CCTGGTGCTCCATGAGGAGAC	CAGACTCTGACCTTTTGCCAGG
USP44	AGGGTGGTCAGGACGTAATA	CCGCGGACAAGTCATAGATAAA
